# Task shifting from general practitioners to practice assistants and nurses in primary care: a cross-sectional survey in 34 countries

**DOI:** 10.1017/S1463423622000470

**Published:** 2022-09-22

**Authors:** Peter Groenewegen, Wienke G.W. Boerma, Peter Spreeuwenberg, Bohumil Seifert, Willemijn Schäfer, Ronald Batenburg, Lilian van Tuyl

**Affiliations:** 1 NIVEL (Netherlands Institute for Health Services Research), Utrecht, The Netherlands; 2 Department of Sociology and Department of Human Geography, Utrecht University, Utrecht, The Netherlands; 3 Institute of General Practice, Charles University, Prague, Czech Republic; 4 Feinberg School of Medicine, Department of Surgery, Northwestern University, Chicago, IL 60611, USA; 5 Department of Sociology, Radboud University, Nijmegen, The Netherlands

**Keywords:** general practice, international comparison, practice nurse, primary care, task shifting

## Abstract

**Aim:**

To describe variation in task shifting from GPs to practice assistants/nurses in 34 countries and to explain differences by analysing associations with characteristics of the GPs and their practices and features of the health care systems.

**Background:**

Redistribution of tasks and responsibilities in primary care are driven by changes in demand, such as the growing number of patients with chronic conditions, and workforce developments, including staff shortage. The need to manage an expanding range of services has led to adaptations in the skill-mix of primary care teams. These developments are hampered by barriers between professional domains.

**Methods:**

Data were collected between 2011 and 2013 through a cross-sectional survey among approximately 7,200 general practitioners (GPs) in 34 countries. Task shifting is measured through a composite score of GPs’ self-reported shifting of tasks. Independent variables at GP and practice level are as follows: innovativeness; part-time working; availability of staff; location and population of the practice. Country-level independent variables are as follows: demand for and supply of care, nurse prescribing, and professionalisation of practice assistants/nurses. Multilevel analysis is used to account for clustering of GPs in countries.

**Findings:**

Countries vary in the degree of task shifting. Regarding GP and practice characteristics, use of electronic health records and availability of support staff in the practice are positively associated with task shifting and GPs’ working hours negatively, in line with our hypotheses. Age of the GPs is, contrary to our hypothesis, positively related to task shifting. These variables explain 11% of the variance at GP level. Two country variables are related to task shifting: a lower percentage of practices without support staff in a country and nurse prescribing rights coincide with more task shifting. The percentage of practices without support staff has the strongest relationship, explaining 73% of the country variation.

## Introduction

Over the past decades, changes in demand for care led to reconsiderations and redistributions of tasks and responsibilities in the primary care workforce across Europe. Primary care practices adapted to the changed patterns of morbidity and patients’ increasingly complex health care needs by, for example, expanding the services offered and the skill-mix of health care workers involved (Van Schalkwyk *et al*., 2020). European primary care services particularly expanded in the area of (chronic) disease management (Schäfer *et al.*, 2016). The adaptations are also visible in the workforce composition of primary care practices, which is increasingly made up of multidisciplinary teams, rather than individual general practitioners (GPs) only (Groenewegen *et al.*, 2015). Such teams enable the redistribution or delegation of tasks to professional support functions. Drivers of these developments were not only changes in the demand for health care, but also the need for increased efficiency, cost containment and difficulties in many countries to attract and retain GPs. Ageing GP populations in Europe and a lack of newly trained GPs have resulted in shortages, in particular in rural areas (Groenewegen *et al.*, 2020).

In this article, we describe and explain the extent of task shifting between GPs and nurses and support personnel in 34 countries. We define task shifting as the reallocation and redistribution of tasks and the sharing of roles among health professions and different groups of health professionals (WHO, 2006; EC, 2019). The number and diversity of staff present in a practice or health centre determines the options for task shifting but may also be a result of this process. In a strictly single-handed GP practice (i.e., without any supporting staff), for example, possibilities for task shifting are absent.

Occupational titles (and the related professional education) strongly differ between countries (Hewko *et al.*, 2015; Schäfer *et al.*, 2018; Kroezen *et al.*, 2018). Therefore, it is easier to identify and label functions carried out in practices than to allocate occupational titles to those executing them. In primary care, assistants are usually called practice assistants or practice secretaries. Nurses in primary care practices may have the occupational title of practice nurse, which does not necessarily imply they are registered nurses. In some countries, nurses working in primary care may functionally be equivalent to practice secretaries or assistants in other countries. Throughout this paper, we will use the term ‘practice assistant/nurse’, and this includes practice secretaries as well.

There is a broad range of tasks that can be shifted to practice assistants/nurses, including routine checks (e.g., blood pressure measurement and health assessments), prescribing drugs and referring patients, more technical procedures (e.g., wound care and removing sutures), and health promotion activities (e.g., patient education on quitting smoking) (Vail *et al.*, 2011; Maier and Aiken, 2016). Previous studies found that for curative services shifted from GPs to nurses, there was no difference in the quality of care provided (Laurant *et al.*, 2007; Martinez-Gonzalez *et al.*, 2015; Lovink *et al.*, 2017; Laurant *et al.*, 2018). However, less is known about task shifting from doctors to nurses in the area of prevention and health education (Laurant *et al.*, 2018).

Some countries have a much longer tradition of task shifting than others. In the UK and the Netherlands, task shifting to practice assistants/nurses in primary care started as early as the 1980s (Van Tuyl *et al.*, 2020), while in other countries, like Belgium (Groenewegen *et al.*, 2015) the dominance of small single-handed GP practices has hampered task shifting. More in general, barriers and facilitators may be sought in the degree of acceptance of task shifting among patients as well as health care professionals, in the organisation and resources of the practices and in regulation and other conditions at country or health system level (Maier and Aiken, 2016; Maier *et al.*, 2017; van der Biezen *et al.*, 2017; Nuttall, 2018; Karimi-Shahanjarini *et al.*, 2019). Therefore, we expect to find large variation in task shifting between the countries included in our study.

In this article, we report on a secondary analysis of the QUALICOPC study, involving a survey among GPs in 34 (mainly) European countries, conducted in 2012. We will first describe the extent of task shifting in these countries. Next, to understand potential barriers and facilitators to task shifting, we will analyse the associations between the extent of task shifting and a number of characteristics of the GPs and their practices and the health care systems in which they operate.

## Hypotheses

Based on theories on readiness for change in general (Weiner, 2009) and on studies on barriers to implementation of task shifting in particular (Niezen and Mathijssen, 2014; Karimi-Shahanjarini *et al.*, 2019), we developed a number of hypotheses on relationships, which are not necessarily causal. We expect that readiness for task shifting is influenced at the following three levels:

- level 1: the *individual* GPs and other professionals involved (e.g., their commitment; efficacy in bringing about task shifting);
- level 2: the *practices* they work in (e.g., experienced urgency of change in skill-mix, available resources in the practice);
- level 3: the *country* or health system (e.g., urgency of task shifting as experienced at policy level; resources made available for this change; adaptation of costing of skill-mix changes).

Influences at different levels may independently increase or decrease the readiness for task shifting, but they are also expected to influence each other. Commitment of care providers, for instance, may be impacted by the experienced urgency of change, and their ability to bring about change depends on the resources available in the primary care practice (Weiner, 2009).

Likewise, potential barriers and facilitators for task shifting can be identified at these three levels:

- level 1: *individual* GPs and practice assistants/nurses (e.g., their views on professional boundaries; knowledge and capabilities);
- level 2: the *practice* environment (e.g., patients’ preferences for a care provider, their acceptance of receiving care from practice assistants/nurses, their knowledge about and trust in practice assistants/nurses’ work);
- level 3: the *country* and health system context (e.g., degree of policy support for task shifting; financial incentives; legal barriers for task shifting; positioning of professional associations; local or regional labour market shortages).

To identify the barriers at patient, GP and practice level, multilevel data are needed at the professional and practice level. For level 3 barriers, data are needed at the health system or country-level influencing policies and policy options. As described below, the QUALICOPC study provides the required multilevel data to analyse the impact of these barriers in an integrative manner.

We will test the following hypotheses:

*At the level of GPs*:

1. More innovative GPs have shifted more tasks to practice assistants/nurses.
  Task shifting can be considered an innovation in the work organisation of general practices. We assume that younger GPs are more willing and capable to adopt innovations in their practices, as shown in the literature about the uptake of electronic health records (EHRs) (Xierali *et al.*, 2013). Following this line of reasoning, it is be expected that GPs, who are more innovative in the use of information technology and systems in their practice, are more inclined to adopt task shifting.
2. *Part-time working GPs have shifted more tasks to practice assistants/nurses.*
  GPs working part-time are assumed to put more effort in maintaining continuity of care (Karimi-Shahanjarini *et al.*, 2019). Working part-time is a challenge to continuity, because GPs are not always available for their patients during usual office hours. To maintain continuity of care during periods of absence, targeted efforts are needed to shift specific care tasks either to other GPs or to supporting staff. As female GPs more frequently work part-time (Van Hassel, 2020), we expect female GPs to have shifted more tasks to practice assistants/nurses than male GPs.
3. *At the level of practices*:
4. *Task shifting by GPs to practice assistants/nurses occurs to a larger extent in GP practices with more supporting staff.*
  Availability of staff enables task shifting; in the absence of support staff, it is not possible. So, the availability of supporting staff is a condition for task shifting. But still, given available supporting staff, we expect variation in the *level* of task shifting, as shifting requires teamwork (Van Tuyl *et al.*, 2020).
5. *Task shifting by GPs to practice assistants/nurses will occur to a larger extent in GP practices with a patient population with higher demands for care and more complex care needs*.
  Following Niezen and Mathijssen (2014), we expect that practices with relatively many patients that have more complex care needs (for example elderly) and practices located in rural areas (e.g., with ageing population or in under-served areas) or inner cities (with problems of deprivation) are faced with relatively higher workloads and will use task shifting to cope with these conditions.
6. *At the level of countries/health systems*:
7. *Task shifting by GPs to practice assistants/nurses will occur to a larger extent in countries where institutional facilitators outweigh barriers for task shifting*
  Task shifting can be strongly influenced by legal and regulatory barriers and facilitators (Van Schalkwyk *et al.*, 2020). In the Czech Republic, for example, GPs are required to employ a nurse, while in the Netherlands the costs of employing a practice nurse are formally reimbursed (Van Tuyl *et al.*, 2020).
8. *Task shifting by GPs to practice assistants/nurses will occur to a larger extent in countries with strongly ageing populations and/or lower or decreasing numbers of GPs per capita.*
  In such countries, policy-makers may feel more urgency to promote task shifting, which will drive the employment of practice assistants/nurses and task shifting within practices.
9. *Task shifting by GPs to practice assistants/nurses will occur to a lesser extent in countries where professional boundaries between GPs and supporting and nursing staff are relatively strict.*
  Professional boundaries are particularly important in strongly organised occupations, such as medical doctors (Abbott, 1988). Shared views on professional boundaries may overrule GPs’ individual attitudes and willingness to shift tasks as well as popular trust in the capabilities of practice assistants/nurses to take on tasks (Van Tuyl *et al.*, 2020). As the guards of professional boundaries, professional associations have a keen interest in task shifting issues (see Kroezen *et al.*, 2011).
10. *Task shifting by GPs to practice assistants/nurses will occur to a larger extent in countries where professionalisation of practice assistants/nurses is more advanced.*
  As a counterforce to the position of medical associations, professional associations of practice assistants/nurses have a role in the promotion of task shifting. The more professionalised practice assistants/nurses are, the more task shifting will occur in primary care. Indicators for the professionalisation of practice nurses/assistants are, for example, the establishment of a professional association and education of practice assistants/nurses (Kroezen *et al.*, 2018; Van Tuyl *et al.*, 2020).

## Data and methods

### The QUALICOPC study

Data were collected between 2011 and 2013 from approximately 7,200 GPs in 31 European countries (EU 26 – except France –, and Iceland, Norway, North Macedonia, Turkey, Switzerland and England) and three non-European countries (Canada, New Zealand and Australia). In each country, a sample of around 220 GPs completed a questionnaire, except for small countries (Cyprus, Iceland, Luxembourg and Malta) where this was around 75. In most countries, a random sample of GPs was invited to participate. In countries without a national sampling frame, alternatives were sought as close as possible to a random sample. Only one GP per practice participated in the study. The participation rates varied from less than 10% in Austria and Belgium to over 70% in Malta and Spain, with an average of 30% (Groenewegen *et al.*, 2016).

Details of the study design and the development of the questionnaire can be found elsewhere (Schäfer *et al.*, 2011, Schäfer *et al.*, 2013). Ethical review was conducted in accordance with the legal requirements in each country (Rotar Pavlic *et al.*, 2015).

### Measures

#### Dependent variable

The degree of task shifting was measured through a sum score of GPs’ responses to the following questions on four different tasks: ‘Does your practice nurse or assistant independently provide: 1. Immunisation; 2. Health promotion; 3. Routine checks of chronically ill patients; 4. Minor procedures?’ Answering options were: ‘yes’ (counted as 1), and ‘no’ or ‘not applicable (no nurse in my practice)’ (counted as 0). Therefore, the composite score ranges between 0 and 4. We combined the categories ‘no’ and ‘not applicable’ (having no nurse or assistant to delegate tasks to, amounts to the same as not delegating these tasks).

#### Independent variables at GP and practice level

##### Innovativeness (hypothesis 1)

As a first indicator for innovativeness, we used the number of EHRs applications used by GPs (De Rosis & Seghieri, 2015). In the survey, GPs could select the following options (multiple answers possible): ‘not applicable (I don’t use a computer)’; making appointments; issuing invoices; issuing medicine prescriptions; keeping records of consultations; sending referral letters to medical specialists; searching medical information on the Internet; storing diagnostic test results; and sending prescriptions to the pharmacy. The answers were combined into a sum score, ranging from 0 (no computer use) to 8 (applying all EHR applications listed). Besides, as a second proxy for innovativeness we used GPs’ age, assuming that younger GPs are more trained and familiar with using EHR applications.

##### Part-time working (hypothesis 2)

We do not have a direct measure of GPs’ part-time working status. Instead, we used the GPs reported weekly workhours and added the average in a country as an offset in the statistical analysis.

##### Availability of staff (hypothesis 3)

Availability of staff at GP and practice level was measured through two variables: availability of support and nursing staff (yes/no – receptionist/assistant, practice nurse, home care nurse or nurse practitioner); and other professionals (yes/no – other professionals in the practice).

##### Practice location and population (hypothesis 4)

Information on the practice location was derived from the answer on the question: How would you characterise the place where you are currently practising? (possible answers: big (inner)city, suburbs, (small) town, mixed urban–rural and rural). The practice composition was measured as the estimated proportion of elderly people; people from ethnic minorities; and deprived people (possible answers: above average, average and below average).

#### Independent variables at country level

##### Institutional environment (hypothesis 5)

In the absence of direct information to operationalise barriers or facilitators in the institutional environment of primary care practices, we assume that the institutional environment is more facilitating when it is more usual to have support staff in the practice. We therefore aggregated the number of practices without receptionist/assistant, practice nurse, home care nurse or nurse practitioner to country level.

##### Demand for and supply of primary care (hypothesis 6)

For demand and supply of primary care, we used the following three indicators. Firstly, population ageing, that is, the increase in the percentage of the population over 65 years old between 1993 and 2012 retrieved from World Bank data (source: http://databank.worldbank.org/data/home.-aspx-

Secondly, GP shortages were derived from the PHAMEU framework (Primary Health Care Activity Monitor for Europe; Kringos *et al.*, 2010): Do (regional or national) shortages exist of GPs according to usual national norms? (no shortage = 3; shortage in some regions = 2; nationwide shortage = 1; no info for Ireland and Luxemburg).

Finally, the ageing of GPs was measured by the percentage of GPs over 60 years of age (aggregated from the QUALICOPC data).

##### Professional boundaries between GPs and supporting and nursing staff (hypothesis 7)

As a proxy indicator for professional boundaries, we used data on whether nurses have prescription rights in a country. Using data from Kroezen *et al.* (2011) and Maier (2019), we classified countries into three categories: 1 = no prescription rights (Austria, Belgium, Bulgaria, Czech Republic, Germany, Greece, Hungary, Iceland, Italy, Latvia, Lithuania, Luxemburg, Malta, North Macedonia, Portugal, Rumania, Slovakia, Slovenia and Turkey)); 2 = prescription rights introduced after 2010 (Cyprus, Estonia, Netherlands, Poland, Spain and in one Canton in Switzerland); 3 = prescription rights granted up to 2010 (Australia, Canada, Denmark, Finland, Ireland, New Zealand, Norway, Sweden and UK). The category ‘prescription rights introduced after 2010’ was added because introduction of nurse prescribing is a lengthy process (Maier, 2019), and it is likely that in these countries professional boundaries between nurses and doctors were already under debate in the preceding years.

##### Professionalisation of practice assistants/nurses (hypothesis 8)

A scale consisting of the following indicators derived from the PHAMEU database (Kringos *et al.*, 2010) was used:

- Is there professional training specifically for district or community nurses?(yes/no)
- Is there professional training specifically for PC/GP practice nurses? (Yes/no)
- Do national associations or organisations of PC nurses exist in this country?(Yes/no)
- Is a professional journal on PC nursing being published in this country? (Yes/no).

### Statistical analysis

Multilevel analysis (Leyland, Groenewegen 2020).

The analysis was done using multilevel analysis to account for the nested structure of the data.

We used the random effects (variances) at GP and country level to describe the clustering of task shifting by GPs. The country-level variances were used to construct a caterpillar plot to show the differences between countries on the task shifting scale. The GP, practice and country variables were included in a multilevel linear regression analysis with the scale value as dependent variable.

For the GP and practice characteristics, we used list-wise deletion of missing values. As the number of countries is relatively small for statistical analysis, we included country-level variables one at a time. We use *P* < 0.05 as the boundary value for statistical significance.

The modelling strategy consists of the following steps:

1. empty model to calculate the clustering of the dependent variable within GP practices and countries;
2. adding GP and practice variables and average number of working hours per country;
3. average number of working hours dropped and country variables (one-by-one) added.

Analyses were performed in MLwiN, version 2.30.

### Ethical approval

Ethical approval for the QUALICOPC study was acquired in accordance with the legal requirements in each country (De Rosis and Seghieri, 2015).

## Results

Descriptive data on the independent variables are provided in Supplementary table 1. We distinguish between variables measured at the GP and practice level and variables measured at the country level. Across, all practices and countries, the average number of EHR applications used for clinical purposes was 6 on a scale from 0 to 8. The average age of GPs was 50 years. On average, they worked 40 h per week. Most GPs had nurses/practice assistants or secretaries as support staff and in 11% of the practices also other professionals were active. Nearly one-third of the practices was located in cities. The GPs reported mainly an average share of elderly and socially deprived people in their practice and a lower than average share of people from ethnic minorities.

For variables, measured at the country level, in one-fifth of the countries there was no shortage of GPs. The average percentage of GPs of 60 years and over was 17. In more than half of the countries, nurses had no prescribing rights at the time of the survey and the scale for professionalisation averaged 7.2 on a scale from 4 to 12.

**Figure 1.**
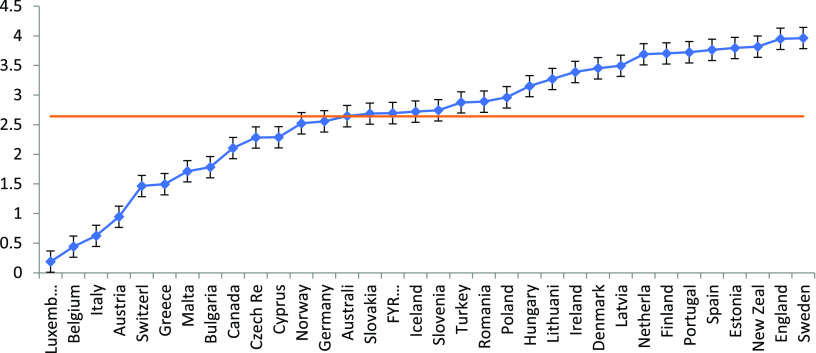
Task shifting scale by country (based on empty model)

Figure 1 shows the distribution of the task shifting scale for the 34 countries in the study. The frequency distributions of the separate items of the scale are in Supplementary tables 2–5. Countries differ in the occurrence of task shifting from GPs to nurses and/or assistants. Task shifting is most common in England, Sweden and New Zealand, while Luxemburg, Belgium and Italy are in the lower end of the distribution. The large variation between countries are also reflected in the intraclass correlation (ICC) which is 44% (Table 1). The ICC is a measure for the extent of clustering of the observations. It shows to what extent task shifting in GP practices looks alike within countries.

**Table 1.** Linear multilevel regression analysis of task shifting in general practice (N_countries_ = 34; n_GPs_ = 6,257)

	Empty model	Model 1: GP and practice variables	Model 2: + country variables (one-by-one)^a^
***Fixed effects***			
Intercept	2.661 (0.178)	2.654 (0.125)	2.655 (0.094)
*GP/practice level*			
Use of EHR applications		0.081 (0.008)***	0.080 (0.008)***
GPs’ age		0.006 (0.002)***	0.006 (0.002)***
Hours worked by GPs		−0.003 (0.001)**	−0.003 (0.001)**
Support staff (Y/N)		0.464 (0.020)***	0.461 (0.020)***
Other professionals (Y/N)		−0.010 (0.013)	−0.010 (0.013)
Practice location (ref. big city)			
- Suburbs		0.137 (0.048)***	0.137 (0.048)***
- Small towns		0.198 (0.041)***	0.200 (0.041)***
- Mixed urban–rural		0.080 (0.045)	0.081 (0.045)
- Rural		0.103 (0.046)**	0.104 (0.046)**
Proportion elderly		−0.019 (0.021)	−0.019 (0.021)
Proportion ethnic minority		−0.027 (0.021)	−0.027 (0.021)
Proportion deprived		0.092 (0.022)***	0.092 (0.022)***
*Country level*			
Average working hours GPs		−0.063 (0.029)**	
% No support staff available			−0.045 (0.008)***
Increase population ≥65			0.058 (0.085)
GP shortage (1 = no shortage – 3 = nationwide shortage)^b^			0.157 (0.123)
GPs over 60 years			−0.011 (0.015)
Nurse prescribing (1 = no prescription rights – 3 = prescription rights)			0.249 (0.150)*
Professionalisation scale^c^			0.068 (0.051)
*Random effects*			
GP/practice variance	1.360 (0.024)	1.207 (0.022)	1.207 (0.022)
Country variance	1.065 (0.261)	0.526 (0.130)	0.292 (0.073)
ICC	43.9	30.3	19.5

*P < 0.10; **P < 0.05; ***P < 0.01.

a Coefficients of GP/practice-level variables and random effects taken from model 3 with the percentage of practices without support as independent variable at country level.

b No information for Ireland, Luxembourg and Canada.

c No information for Malta.

Most of the studied GP and practice variables are significantly related to task shifting (Table 1). GPs who use more different EHR applications have shifted more tasks to nurses and/or assistants, and the same applies to older GPs, GPs who have more support staff in their practice, and GPs with a practice in suburbs, small towns or rural areas, compared to those working in inner cities. GPs who work less hours (controlling for the average number of working hours in each country) have shifted more tasks.

The GP and practice variables explain just over 11% the variance in task shifting at the level of GPs (100 minus [GP variance in model 1 without average working hours at country level, divided by the GP variance in the empty model] times 100).

Regarding the country variables, the percentage of practices without support staff is negatively related to task shifting, while nurse prescribing is positively related to task shifting by GPs. The first shows the strongest relationship and explains 73% of the country variation in task shifting by GPs (100 minus [country variance in model 2 with percentage of practices without support staff, divided by the country variance in the empty model] times 100).

## Discussion

In 2012, task shifting in primary care to nurses/assistants was very common in the 34 countries included in our study. The extent of task shifting by GPs differs between countries with England on the high end of the distribution and Luxemburg on the low end. We tested a number of hypotheses related to GP, practice and country characteristics. The following hypotheses were (partly) confirmed. First, GPs that use more EHR applications in their practice more often shifted tasks to nurses or assistants. We used computer use as an indicator for innovativeness (hypothesis 1). GPs who work less hours (while controlling for the average number of working hours per country) as an indicator for part-time working had more often shifted tasks (hypothesis 2). Hypothesis 4 about patient populations with higher demands for care and more complex care needs was only partly confirmed – only in practices with above average deprived persons task shifting was higher; however, the results for practice location varied and there was rather less task shifting in inner city practices. Hence, it appears that increasing (complexity of) demand for care and (expected future) shortage of GPs are not systematically related to task shifting. Remarkable is the fact that older GPs, contrary to hypothesis 1, have shifted task to a larger extent than younger GPs.

Two of our hypotheses at country level were confirmed. In countries where it is less common to have practice support, task shifting occurs less in the practices that have support staff to shift tasks to. We have used this variable as an indicator for an institutional factor in the absence of direct information on barriers or facilitators in the institutional environment (hypothesis 5). Second, in countries where nurses have prescribing rights, GPs have shifted tasks to nurses/assistants to a greater extent. We have used this as an indicator for less strict professional boundaries (hypothesis 7).

Our first confirmed hypothesis related to innovativeness (Greenhalgh *et al.*, 2004). The use of computers in practice for more different purposes indicates the readiness of GPs and their practices to implement innovations. However, our reasoning that younger GPs are more open to innovations was apparently not correct, as it is not related to the extent of task shifting. A possible explanation may be that older GPs have more insight in the competences of their support staff (who perhaps are also older and more experienced) to take over tasks. Where shifting of particular tasks to practice assistants/nurses is not formally allowed, it is possible that older GPs yet take more liberty to delegate tasks informally. Another explanation may be that older, more experienced GPs increasingly shift their own tasks towards management of their practice, hence shifting tasks related to patient care in the direction of other support staff. However, these explanations should be tested independently. In sum, our analysis suggests that the extent of task shifting is related to innovativeness at GP level and to professional boundaries between nurses and doctors at the country level.

The confirmed hypothesis about professional boundaries relates to the system character of the position of different health care professions and their mutual relations (Abbott, 1988). This shows that task shifting should not be considered in isolation and that it is sensitive to the context. It is part of broader processes of interprofessional domain setting, which are intertwined with the educational system and the development of mutual trust between doctors, practice assistants/nurses and patients (Frenk *et al.*, 2010). Consequently, although the initiative of task shifting will often be within practices, our study shows the importance of a facilitating environment at a system level. We used nurse prescribing rights as an indicator for debates on professional boundaries. Admittedly, these debates do not necessarily lead to less strict boundaries (as we formulated in our hypothesis) but may also lead to new, strict boundaries. In our view, the debate about prescription rights of nurses in itself indicates that change is possible and as such this makes for an environment in which task shifting will be seen as an option. It should be added that nurse prescribing is a form of task shifting; however, we are convinced that we can still use as part of the explanation of task shifting in general practice, because nurse prescribing tends to be introduced in the hospital context first.

At health system level, cost containment may also have played a role in policies that support shifting tasks from GPs to nurses and support personnel. However, there is hardly any information about national policies regarding task shifting, but we know that the value of teamwork and the optimal team skill-mix are considered important policy issues in many countries (Van Schalkwyk *et al.*, 2020). Related to this, we did not have information about the education and skills of practice nurses/assistants employed in the practices in our survey. Most likely these differ between and within countries. From a quality of care perspective, an additional question is how practice nurses/assistants perform the tasks that have been shifted to them and how this differs between GP practices. Systematic reviews have shown that the quality of care performed by nurses is at least as good as care from GPs (Laurant *et al.*, 2007; 2018; Martinez-Gonzalez *et al.*, 2015; Lovink *et al.*, 2017).

Our hypotheses concerning the role of increasing (complexity of) demand for care and (expected future) shortages of GPs on task shifting were not systematically confirmed. Yet, these developments have further progressed in many countries, with a strong impact on the workload and availability of GPs for which task shifting can be among the solutions. This makes insight into barriers and facilitators to task shifting important to pave the way for new initiatives to unfold. Task shifting from GPs to nursing and support staff can be considered as one of the first emerging forms of task shifting. However, also other professionals play increasingly important roles in the strengthening of primary care organisation. Several countries invested in task shifting from GPs to pharmacists. In Canada, New Zealand, the US and the UK, pharmacists have prescribing rights with varying levels of responsibilities. In the Netherlands, experiments with pharmacists as clinical care provider in primary health care teams and employee within a GP practice show promising results in terms of improved safety and effectiveness of pharmacotherapy in primary care, including a reduced risk of medication-related hospitalisations compared to usual care (Sloeserwij *et al.*, 2019).

Task shifting in primary care is a complex and context-dependent phenomenon. This means that direct policy implications of our analysis are difficult to draw. The innovativeness of GPs could be stimulated through their education and through incentives to practicing GPs. Changes in the institutional environment usually take time but could also find a starting point in education; interprofessional education could change the existing barriers between professions. However, the COVID-19 pandemic has shown that under pressure, changes in the tasks of both GPs and support staff have occurred quickly, but the question is of course whether these changes will sustain when the pandemic recedes.

Our analysis and the data that we used have a number of strengths and limitations, some of which were already mentioned. We have data from a large number of countries, that is, 34. This makes a statistical analysis at both GP/practice and country/health system level possible. We applied state-of-the-art statistical analysis that takes the hierarchical character of the data into account. The response rates for the QUALICOPC study differed but averaged around 30%. The samples were as much as possible random samples, but this was not attainable in all countries. We expect that this bias was not strong as the sample distribution by age and sex of GPs was close to the national distribution (Groenewegen *et al.*, 2016). However, as in any survey study, there may be non-response bias. Social desirability might have influenced some of the answers.

We performed a secondary analysis of existing data, not specifically designed to study task shifting. Consequently, the measurements were quite general.

A further limitation is that the data are by now somewhat old (collected 2011–2013). This is particularly relevant for the descriptive value of the study, but our hypothesis testing is less sensitive to this. If our data collection could be repeated as of now, we expect to see effects of different changes over time. For example, there is increased acceptance of task shifting by the population, for example, in Germany (Jedro *et al.*, 2020). Computer use in primary care practices will have increased even further. Prescribing rights of nurses are more prevalent today than at the time of data collection, but we used the formalisation of such prescribing rights in more recent times as an indicator for the debate about professional boundaries which was likely going on when data were collected. We have no data on changes in the aspects of professionalisation of practice nurses/assistants.

Finally, it should be emphasised that the associations we found cannot be considered as causal associations. Interprofessional relations have a system character and complex feedbacks (Abbott, 1988). An implication of the importance of the system level is that there are no easy recipes for introducing task shifting from GPs to practice assistants/nurses in countries where this is not yet prevalent. Simply transferring an innovation from one health care system to another often does not work (Nolte and Groenewegen, 2021). Finally, the absence of information on education, skills and competences, and the quality of task performance has limited the scope of our study.

## Conclusions

Task shifting by GPs to practice assistants/nurses can be an answer to current challenges in primary care. The extent of task shifting in a country strongly depends on a facilitating institutional environment, as indicated by how common it is to have support staff in GP practices and by prescription rights for nurses. Within countries, task shifting is more prevalent in practices with an innovating attitude, with more support staff and among GPs who work less hours and older GPs. Given the importance of task shifting and its potential for innovation (Van Tuyl *et al.*, 2021), we recommend an assessment of changes in this area in the countries studied in a new survey.

## Supplementary material

For supplementary material accompanying this paper visit http://doi.org/10.1017/S1463423622000470. click here to view supplementary material

## Declaration of retraction and revision

The previous version of this paper has been retracted. This is the revised version. The reason is that we have made a serious mistake in recoding the dependent variables that form the task shifting scale. Instead of recoding ‘not applicable (no nurse in my practice)’ into ‘no’, as stated in the method section, we have recoded it by mistake into ‘yes’. As a consequence Figure 1 and Table 1 and the analysis behind them are incorrect. The tables presented in the Supplementary Material are correct. The analysis has been done anew and the conclusions are partly different. For those countries that have a low number of practices with ‘not applicable (no nurse in my practice)’ the differences are small and this is the majority of countries. However, the results and discussion sections have been rewritten.
