# Supplementary material for: Task shifting from general practitioners to practice assistants and nurses in primary care: a cross-sectional survey in 34 countries
Source: Prim Health Care Res Dev. 2022 Sep 22;23:e60. doi: 10.1017/S1463423622000470 (PMC9532851; doi:10.1017/S1463423622000470)
Supplement: Supplementary file 1 [file S1463423622000470sup001.docx]

Supplementary table 1: Descriptive information on the independent variables

|  | Percentage | Mean (standard deviation) | Median (interquartile range) | Number of missing observations |
| --- | --- | --- | --- | --- |
| *GP/practice level* |  |  |  |  |
| Computer use |  | 6.35 (2.29) | 7 (3) | 9 |
| GPs’ age |  | 50.05 (9.66) | 51 (14) | 76 |
| Hours worked |  | 39.9 (10.68) | 40 (10) | 138 |
| Support staff (perc. yes) | 90% |  |  |  |
| Other professionals (perc. yes) | 11% |  |  | 0 |
| Practice location |  |  |  | 82 |
| - big city | 31% |  |  |  |
| - suburbs | 13% |  |  |  |
| - small towns | 22% |  |  |  |
| - mixed urban-rural | 17% |  |  |  |
| - rural | 17% |  |  |  |
| Proportion elderly |  |  |  | 68 |
| - below average | 16% |  |  |  |
| - average | 46% |  |  |  |
| - above average | 38% |  |  |  |
| Proportion ethnic minority |  |  |  | 601 |
| - below average | 56% |  |  |  |
| - average | 28% |  |  |  |
| - above average | 16% |  |  |  |
| Proportion deprived |  |  |  | 460 |
| - below average | 32% |  |  |  |
| - average | 45% |  |  |  |
| - above average | 23% |  |  |  |
| *Country level* |  |  |  |  |
| Average working hours |  | 39.73 (4.37) | 38.61 (3.94) | 0 |
| No availability of support staff |  | 6.25 (12.43) | 0.98 (4.29) | 0 |
| Increase population >=65 |  | 3.06 (1.59) | 3.09 (2.4) | 0 |
| GP shortage |  |  |  | 3 |
| - no shortage | 23% |  |  |  |
| - shortage in some regions | 58% |  |  |  |
| - nation-wide shortage | 19% |  |  |  |
| GPs over 60 yrs |  | 17.27 (8.92) | 16.76 (14.28) | 0 |
| Nurse prescribing |  |  |  | 0 |
| - no prescription rights | 56% |  |  |  |
| - introduced after 2010 | 18% |  |  |  |
| - prescription rights | 26% |  |  |  |
| Professionalisation (WFD scale) |  | 7.19 (2.67) | 8 (4) | 1 |

Supplementary table 2: Does your nurse or assistant independently provide immunisation

| Country | No | Yes | Not applicable | Missing | Total | Yes as % of valid n* |
| --- | --- | --- | --- | --- | --- | --- |
| Austria | 99 | 31 | 52 | 2 | 184 | 17.0 |
| Belgium | 56 | 35 | 308 | 9 | 408 | 8.8 |
| Bulgaria | 95 | 101 | 23 | 4 | 223 | 46.1 |
| Cyprus | 24 | 43 | 4 | 0 | 71 | 60.6 |
| Czech Republic | 17 | 199 | 3 | 0 | 219 | 90.9 |
| Denmark | 13 | 192 | 7 | 0 | 212 | 90.6 |
| Estonia | 10 | 118 | 0 | 1 | 129 | 92.2 |
| Finland | 11 | 272 | 1 | 4 | 288 | 95.8 |
| Germany | 48 | 185 | 1 | 4 | 238 | 79.1 |
| Greece | 31 | 109 | 79 | 1 | 220 | 49.8 |
| Hungary | 65 | 154 | 1 | 2 | 222 | 70.0 |
| Iceland | 8 | 69 | 2 | 1 | 80 | 87.3 |
| Ireland | 12 | 145 | 9 | 3 | 169 | 87.3 |
| Italy | 49 | 32 | 129 | 8 | 218 | 15.2 |
| Latvia | 22 | 190 | 1 | 5 | 218 | 87.6 |
| Lithuania | 54 | 167 | 3 | 1 | 225 | 74.6 |
| Luxembourg | 16 | 3 | 56 | 3 | 78 | 4.0 |
| Malta | 7 | 32 | 30 | 1 | 70 | 46.4 |
| Netherlands | 34 | 197 | 4 | 3 | 238 | 83.8 |
| Norway | 9 | 179 | 8 | 2 | 198 | 91.3 |
| Poland | 46 | 172 | 2 | 0 | 220 | 78.2 |
| Portugal | 5 | 209 | 0 | 2 | 216 | 97.7 |
| Romania | 89 | 130 | 0 | 1 | 220 | 56.4 |
| Slovakia | 22 | 193 | 0 | 5 | 220 | 89.8 |
| Slovenia | 44 | 163 | 0 | 0 | 207 | 78.7 |
| Spain | 10 | 416 | 1 | 1 | 428 | 97.4 |
| Sweden | 1 | 96 | 0 | 0 | 97 | 99.0 |
| Switzerland | 42 | 123 | 33 | 1 | 199 | 62.1 |
| Turkey | 29 | 269 | 1 | 0 | 299 | 90.0 |
| England | 2 | 167 | 0 | 2 | 171 | 98.9 |
| Australia | 25 | 100 | 25 | 2 | 152 | 66.7 |
| Canada | 21 | 136 | 25 | 0 | 182 | 74.7 |
| New Zealand | 1 | 161 | 3 | 3 | 168 | 97.6 |
| FYR Macedonia | 57 | 78 | 8 | 0 | 143 | 54.5 |
| Total | 1.074 | 4.866 | 819 | 71 | 6.830 | 72.0 |

* Valid n is yes, no and not applicable.

Supplementary table 3: Does your nurse or assistant independently provide health promotion

| Country | No | Yes | Not applicable | Missing | Total | Yes as % of valid n* |
| --- | --- | --- | --- | --- | --- | --- |
| Austria | 91 | 39 | 52 | 2 | 184 | 21.4 |
| Belgium | 47 | 44 | 309 | 8 | 408 | 11.0 |
| Bulgaria | 66 | 126 | 24 | 7 | 223 | 58.3 |
| Cyprus | 38 | 29 | 4 | 0 | 71 | 40.8 |
| Czech Republic | 77 | 136 | 3 | 3 | 219 | 63.0 |
| Denmark | 24 | 179 | 9 | 0 | 212 | 84.4 |
| Estonia | 1 | 127 | 0 | 1 | 129 | 98.4 |
| Finland | 10 | 272 | 1 | 5 | 288 | 96.1 |
| Germany | 142 | 87 | 1 | 8 | 238 | 37.6 |
| Greece | 79 | 61 | 79 | 1 | 220 | 27.7 |
| Hungary | 33 | 184 | 1 | 4 | 222 | 84.4 |
| Iceland | 22 | 55 | 2 | 1 | 80 | 69.6 |
| Ireland | 7 | 150 | 9 | 3 | 169 | 90.4 |
| Italy | 43 | 36 | 131 | 8 | 218 | 17.1 |
| Latvia | 16 | 200 | 1 | 1 | 218 | 92.2 |
| Lithuania | 14 | 208 | 3 | 0 | 225 | 92.4 |
| Luxembourg | 17 | 2 | 56 | 3 | 78 | 2.7 |
| Malta | 7 | 31 | 31 | 1 | 70 | 44.9 |
| Netherlands | 5 | 225 | 4 | 4 | 238 | 96.2 |
| Norway | 96 | 92 | 8 | 2 | 198 | 46.9 |
| Poland | 48 | 169 | 2 | 1 | 220 | 77.2 |
| Portugal | 12 | 200 | 0 | 4 | 216 | 91.3 |
| Romania | 34 | 186 | 0 | 0 | 220 | 84.5 |
| Slovakia | 56 | 158 | 0 | 6 | 220 | 73.8 |
| Slovenia | 61 | 146 | 0 | 0 | 207 | 70.5 |
| Spain | 19 | 406 | 1 | 2 | 428 | 95.3 |
| Sweden | 0 | 97 | 0 | 0 | 97 | 100.0 |
| Switzerland | 134 | 30 | 33 | 2 | 199 | 15.2 |
| Turkey | 59 | 239 | 1 | 0 | 299 | 79.9 |
| England | 0 | 168 | 0 | 3 | 171 | 100.0 |
| Australia | 24 | 100 | 25 | 3 | 152 | 67.1 |
| Canada | 43 | 110 | 28 | 1 | 182 | 60.8 |
| New Zealand | 1 | 163 | 3 | 1 | 168 | 97.6 |
| FYR Macedonia | 37 | 100 | 6 | 0 | 143 | 69.9 |
| Total | 1.363 | 4.555 | 827 | 85 | 6.830 | 67.5 |

Supplementary table 4: Does your nurse or assistant independently provide routine checks of chronically ill patients

| Country | No | Yes | Not applicable | Missing | Total | Yes as % of valid n* |
| --- | --- | --- | --- | --- | --- | --- |
| Austria | 70 | 60 | 52 | 2 | 184 | 33.0 |
| Belgium | 43 | 49 | 308 | 8 | 408 | 12.2 |
| Bulgaria | 143 | 46 | 24 | 10 | 223 | 21.7 |
| Cyprus | 24 | 43 | 4 | 0 | 71 | 60.6 |
| Czech Republic | 147 | 62 | 3 | 7 | 219 | 29.2 |
| Denmark | 30 | 174 | 8 | 0 | 212 | 82.1 |
| Estonia | 10 | 119 | 0 | 0 | 129 | 92.2 |
| Finland | 40 | 241 | 1 | 6 | 288 | 85.5 |
| Germany | 67 | 164 | 1 | 6 | 238 | 70.7 |
| Greece | 62 | 78 | 79 | 1 | 220 | 35.6 |
| Hungary | 43 | 177 | 1 | 1 | 222 | 80.1 |
| Iceland | 50 | 27 | 2 | 1 | 80 | 34.2 |
| Ireland | 36 | 121 | 9 | 3 | 169 | 71.6 |
| Italy | 41 | 35 | 133 | 9 | 218 | 16.7 |
| Latvia | 40 | 173 | 1 | 4 | 218 | 80.8 |
| Lithuania | 63 | 159 | 3 | 0 | 225 | 70.7 |
| Luxembourg | 17 | 2 | 56 | 3 | 78 | 2.7 |
| Malta | 10 | 27 | 32 | 1 | 70 | 39.1 |
| Netherlands | 1 | 230 | 4 | 3 | 238 | 97.9 |
| Norway | 151 | 38 | 8 | 1 | 198 | 19.3 |
| Poland | 58 | 159 | 2 | 1 | 220 | 72.6 |
| Portugal | 23 | 191 | 0 | 2 | 216 | 89.3 |
| Romania | 72 | 148 | 0 | 0 | 220 | 67.3 |
| Slovakia | 111 | 101 | 0 | 8 | 220 | 47.6 |
| Slovenia | 116 | 90 | 0 | 1 | 207 | 43.7 |
| Spain | 40 | 386 | 1 | 1 | 428 | 90.4 |
| Sweden | 0 | 97 | 0 | 0 | 97 | 100.0 |
| Switzerland | 124 | 39 | 34 | 2 | 199 | 19.6 |
| Turkey | 185 | 113 | 1 | 0 | 299 | 37.8 |
| England | 0 | 169 | 0 | 2 | 171 | 100.0 |
| Australia | 35 | 89 | 25 | 3 | 152 | 59.7 |
| Canada | 75 | 78 | 29 | 0 | 182 | 42.9 |
| New Zealand | 14 | 150 | 3 | 1 | 168 | 89.8 |
| FYR Macedonia | 46 | 93 | 4 | 0 | 143 | 65.0 |
| Total | 1.987 | 3.928 | 828 | 87 | 6.830 | 58.3 |

Supplementary table 5: Does your nurse or assistant independently provide minor procedures( e.g. ear syringing, wound treatment)

| Country | No | Yes | Not applicable | Missing | Total | Yes as % of valid n* |
| --- | --- | --- | --- | --- | --- | --- |
| Austria | 90 | 40 | 52 | 2 | 184 | 22.0 |
| Belgium | 48 | 45 | 308 | 7 | 408 | 11.2 |
| Bulgaria | 70 | 121 | 24 | 8 | 223 | 56.3 |
| Cyprus | 20 | 47 | 4 | 0 | 71 | 66.2 |
| Czech Republic | 110 | 102 | 3 | 4 | 219 | 47.4 |
| Denmark | 14 | 189 | 9 | 0 | 212 | 89.2 |
| Estonia | 3 | 126 | 0 | 0 | 129 | 97.7 |
| Finland | 2 | 281 | 1 | 4 | 288 | 98.9 |
| Germany | 71 | 162 | 1 | 4 | 238 | 69.2 |
| Greece | 62 | 78 | 79 | 1 | 220 | 35.6 |
| Hungary | 36 | 184 | 1 | 1 | 222 | 83.3 |
| Iceland | 13 | 64 | 2 | 1 | 80 | 81.0 |
| Ireland | 9 | 148 | 9 | 3 | 169 | 89.2 |
| Italy | 46 | 30 | 133 | 9 | 218 | 14.4 |
| Latvia | 25 | 188 | 1 | 4 | 218 | 87.9 |
| Lithuania | 20 | 202 | 3 | 0 | 225 | 89.8 |
| Luxembourg | 15 | 4 | 56 | 3 | 78 | 5.3 |
| Malta | 10 | 27 | 32 | 1 | 70 | 39.1 |
| Netherlands | 16 | 215 | 4 | 3 | 238 | 91.5 |
| Norway | 1 | 187 | 8 | 2 | 198 | 95.4 |
| Poland | 56 | 161 | 2 | 1 | 220 | 73.5 |
| Portugal | 10 | 204 | 0 | 2 | 216 | 95.3 |
| Romania | 49 | 169 | 0 | 2 | 220 | 77.5 |
| Slovakia | 89 | 124 | 0 | 7 | 220 | 58.2 |
| Slovenia | 39 | 168 | 0 | 0 | 207 | 81.2 |
| Spain | 23 | 404 | 1 | 0 | 428 | 94.4 |
| Sweden | 1 | 96 | 0 | 0 | 97 | 99.0 |
| Switzerland | 68 | 95 | 34 | 2 | 199 | 48.2 |
| Turkey | 59 | 239 | 1 | 0 | 299 | 79.9 |
| England | 1 | 168 | 0 | 2 | 171 | 99.4 |
| Australia | 18 | 107 | 25 | 2 | 152 | 71.3 |
| Canada | 21 | 132 | 29 | 0 | 182 | 72.5 |
| New Zealand | 1 | 163 | 3 | 1 | 168 | 97.6 |
| FYR Macedonia | 40 | 101 | 2 | 0 | 143 | 70.6 |
| Total | 1.156 | 4.771 | 827 | 76 | 6.830 | 70.6 |
